# Normalization of Pain-Evoked Neural Responses Using Spontaneous EEG Improves the Performance of EEG-Based Cross-Individual Pain Prediction

**DOI:** 10.3389/fncom.2016.00031

**Published:** 2016-04-13

**Authors:** Yanru Bai, Gan Huang, Yiheng Tu, Ao Tan, Yeung Sam Hung, Zhiguo Zhang

**Affiliations:** ^1^School of Chemical and Biomedical Engineering, Nanyang Technological UniversitySingapore, Singapore; ^2^School of Data and Computer Science, Sun Yat-Sen UniversityGuangzhou, China; ^3^Department of Electrical and Electronic Engineering, The University of Hong KongHong Kong, Hong Kong

**Keywords:** pain-evoked EEG, spontaneous EEG, normalization, cross-individual prediction, pain prediction

## Abstract

An effective physiological pain assessment method that complements the gold standard of self-report is highly desired in pain clinical research and practice. Recent studies have shown that pain-evoked electroencephalography (EEG) responses could be used as a readout of perceived pain intensity. Existing EEG-based pain assessment is normally achieved by cross-individual prediction (i.e., to train a prediction model from a group of individuals and to apply the model on a new individual), so its performance is seriously hampered by the substantial inter-individual variability in pain-evoked EEG responses. In this study, to reduce the inter-individual variability in pain-evoked EEG and to improve the accuracy of cross-individual pain prediction, we examined the relationship between pain-evoked EEG, spontaneous EEG, and pain perception on a pain EEG dataset, where a large number of laser pulses (>100) with a wide energy range were delivered. Motivated by our finding that an individual's pain-evoked EEG responses is significantly correlated with his/her spontaneous EEG in terms of magnitude, we proposed a normalization method for pain-evoked EEG responses using one's spontaneous EEG to reduce the inter-individual variability. In addition, a nonlinear relationship between the level of pain perception and pain-evoked EEG responses was obtained, which inspired us to further develop a new two-stage pain prediction strategy, a binary classification of low-pain and high-pain trials followed by a continuous prediction for high-pain trials only, both of which used spontaneous-EEG-normalized magnitudes of evoked EEG responses as features. Results show that the proposed normalization strategy can effectively reduce the inter-individual variability in pain-evoked responses, and the two-stage pain prediction method can lead to a higher prediction accuracy.

## Introduction

Pain is an unpleasant experience related to substantive or potential tissue damage (Loeser and Treede, 2008; Brown et al., 2011). Self-report is the gold standard to determine the presence, absence, and the degree of pain perception in clinic practice (Cruccu et al., 2010; Haanpää et al., 2011), but it may fail in certain patient populations, e.g., patients who suffer from consciousness disorders or are in coma (Schnakers and Zasler, 2007). Lack of accurate pain assessment in these populations can lead to inadequate or suboptimal treatment of pain. Therefore, it is of high importance to develop a physiology-based pain assessment method that is independent of participants' subjective rating (Brown et al., 2011; Terhaar et al., 2011; Huang et al., 2013b).

As a sensory perception that involves a complex set of brain activities, pain has been under intensive investigations using brain imaging techniques, such as electroencephalography (EEG) and functional magnetic resonance imaging (fMRI). A variety of neural signatures of pain have been identified from brain imaging data, and the possibility to utilize pain-related neural signatures for pain assessment has been explored in many studies (Bromm and Treede, 1983; Iannetti et al., 2003; Marquand et al., 2010; Brown et al., 2011; Brodersen et al., 2012; Schulz et al., 2012; Zhang et al., 2012). For example, a support vector machine (SVM) trained on fMRI data was verified to be possible for pain assessment (Brown et al., 2011). Schulz et al. also applied a multivariate pattern analysis to predicted individual's pain sensitivity using single-trial pain-related EEG (Schulz et al., 2012).

Particularly, EEG-based pain assessment has attracted a growing interest in recent years, not only because the EEG technique is cheap, easy-to-use, and non-invasive, but also because the relationship between EEG responses and pain perception has been relatively well recognized. In basic research of pain, EEG activities elicited by nociceptive laser heat pulses are widely used to assess neural processing of nociceptive pain (Bromm and Treede, 1983; Iannetti et al., 2003; Treede et al., 2003). A positive relationship between the intensity of perceived pain and a variety of components (such as N2 and P2) in laser-evoked EEG responses has been well documented (Kakigi et al., 1989; Bromm and Treede, 1990; Beydoun et al., 1993; Arendt-Nielsen, 1994; Garcí-Larrea et al., 1997; Iannetti et al., 2005; Huang et al., 2013a). Based on the existing knowledge on relationship between laser-evoked EEG and pain, we have developed a method to predict the level of subjective pain perception using single-trial laser-evoked EEG potentials (Huang et al., 2013a), and achieved good predictive accuracy.

Pain prediction using evoked EEG can be realized at two levels: within-individual pain prediction (the classifier and the prediction model were trained on and applied to the same group of individuals) and cross-individual prediction (the classifier and the prediction model were trained on a group of individuals but applied to different individuals). Since within-individual pain prediction requires real pain ratings for new individuals and is not applicable to people who are unable to reliably express their pain perception (Brodersen et al., 2012; Schulz et al., 2012), cross-individual pain prediction is more desired in clinical uses. However, our previous work (Huang et al., 2013b) showed that cross-individual pain prediction has a significantly lower performance than within-individual prediction, mainly because of the inherent inter-individual variability in pain perception and neural responses. Therefore, incorporating individual factors that are particularly related to inter-individual variability of pain perception or pain-evoked neural activities into the cross-individual pain prediction model is crucial in pain assessment and pain therapy (Davis, 2011), and it is also the objective of the present study.

In an EEG-based pain prediction model that links EEG signals and subjective pain ratings, substantial inter-individual variability is involved in both EEG and ratings. However, it is difficult to improve the performance of cross-individual pain prediction by means of reducing the inter-individual variability of subjective pain ratings, because pain ratings for a new individual, as the unknown variables to be predicted, are not accessible. Therefore, this study is focused exclusively on decreasing the inter-individual variability of pain-related EEG responses: we aim to explore how the pain-related EEG responses vary between individuals at different levels of pain and how to normalize pain-related EEG responses across individuals for a more accurate EEG-based cross-individual pain prediction.

In the present study, we hypothesize that an individual's spontaneous EEG activity can be used to normalize his/her pain-evoked EEG responses so as to improve the accuracy of cross-individual pain prediction. This hypothesis is induced by strong and consistent evidence showing that the magnitudes of a variety of pain-evoked EEG responses are highly correlated with that of spontaneous EEG of the same individual. Actually, the magnitudes of both spontaneous and pain-evoked EEG activities are altered by the difference in individual-specific factors, such as cortical anatomy (e.g., the thickness of the skin and skull) and experimental conditions (e.g., electrode position and scale-electrode impedances; Klistorner and Graham, 2001; You et al., 2012). Therefore, the magnitude of spontaneous EEG has the potential to serve as an individual scale to normalize the magnitude of pain-evoked EEG responses for a reduced inter-individual variability. The normalized magnitudes of pain-evoked EEG are used as features in the subsequent pain prediction. Next, a two-stage cross-individual pain prediction method is developed: a binary classifier to discriminate low-pain (NRS ≤ 4) and high-pain (NRS > 4) followed by a linear prediction model to predict the pain ratings (4–10) for high-pain trials only. The results showed that the proposed spontaneous EEG based normalization can effectively decrease the inter-individual variability in the classifiers and prediction models, and consequently, can increase the accuracy of pain prediction, as compared with the prediction based on raw pain-evoked EEG responses.

## Materials and methods

### Participants

Thirty-four healthy volunteers (17 females and 17 males), aged 18–25 years (Mean ± SD: 21.6 ± 1.7), without a history of chronic pain, participated in the study. All volunteers gave their written informed consent and were paid for their participation. The experiment procedures were approved by the local ethics committee. Before the experiment, they were familiarized with the experimental setup and task.

### Experimental design

Nociceptive-specific radiant-heat stimuli were generated by an infrared neodymium yttrium aluminiumperovskite (Nd:YAP) laser with a wavelength of 1.34 μm (Electronical Engineering, Italy). At this wavelength, laser pulses activate directly nociceptive terminals in the most superficial skin layers. The laser beam was transmitted via an optic fiber and its diameter was set at ~ 7 mm (≈38 mm^2^) by focusing lenses. Laser pulses were directed at the medial side of the dorsum of left hand, between the first and third metacarpus. A He-Ne laser pointed to the area to be stimulated. The duration of the laser pulse was fixed at 4 ms. After each stimulus, the target of the laser beam was shifted by more than 1 cm in a random direction, to avoid nociceptor fatigue or sensitization.

Participants were asked to report the intensity of perceived pain elicited by the laser stimulus, using a numerical rating scale (NRS) ranging from 0 (no pain) to 10 (pain as bad as it could be). Prior to EEG data collection, the highest energy of the laser stimulation, used in the following experiment, was individually determined using the method of limits (from 1 J in step of 0.25 J) until a rating of 8 was reached. No withdrawal reflexes and motor contractions were observed until the stimulation intensity increased to 3.75–4.5 J. During the EEG data collection, 12–15 different levels of laser stimulation energies (from 1 to 3.75–4.5 J, in step of 0.25 J) were adopted, and 10 laser pulses at each energy level, for a total of 120–150 pulses, were delivered in two blocks. Before each block, the surface temperatures of hand dorsum for each participant were measured using an infrared thermometer. The order of stimulus energies was pseudo-randomized. The inter-stimulus interval (ISI) varied randomly between 10 and 15 s (uniformly distributed). An auditory tone delivered between 3 and 6 s after the laser pulse (uniformly distributed) prompted the participants to rate the intensity of pain. The dataset with 12–15 levels of stimulation energy enables a more comprehensive and detailed investigation of the relationship between pain-evoked EEG responses and spontaneous EEG activities and the relationship between pain-evoked EEG responses and subjective pain ratings.

### EEG recording

Participants were seated in a comfortable chair in a silent, temperature-controlled room. They wore protective goggles and were asked to focus their attention on the stimuli and relax their muscles. The EEG data were recorded using a 64-channel EEG cap with Ag-AgCl scalp electrodes placed according to the international 10–20 system (Brain Products GmbH, Munich, Germany; pass band: 0.01–100 Hz; sampling rate: 1000 Hz). The nose was used as the reference electrode, and the impedances of all electrodes were kept lower than 10 kΩ. Electrooculographic (EOG) signals were simultaneously recorded using surface electrodes to monitor ocular movements and eye blinks.

### EEG data analysis

#### Preprocessing

Continuous EEG data from Cz channel were band-pass filtered between 1 and 30 Hz. EEG epochs were extracted using a window analysis time of 1 s (from 0.5 s pre-stimulus to 0.5 s post-stimulus), and baseline corrected using the pre-stimulus interval (−0.5 to 0 s). Artifacts due to eye blinks or eye movements were subtracted using independent component analysis. In all datasets, the independent components which had a large EOG channel contribution and a frontal scalp distribution were removed. The above EEG data preprocessing were realized using EEGLAB (Delorme and Makeig, 2004), an open source toolbox running in MATLAB environment.

#### Feature extraction

EEG trials recorded at Cz (nose referenced) were used for prediction of pain perception. Each EEG trial consists of two segments: the pre-stimulus trial (−0.5 to 0 s) is spontaneous EEG (sEEG) and the post-stimulus trial (0–0.5 s) is dominated by pain-evoked EEG (pEEG) or, more precisely, Aδ-fiber pain-evoked EEG responses. The magnitude of sEEG or pEEG trial is quantified by root mean square (*RMS*)

(1) $$\begin{array}{l} RMS=\sqrt{\frac{1}{K}\overset{K}{\underset{k=1}{\sum }}x_{k}^{2}}, \end{array}$$

where *x*_*k*_ is the *k*-th sample of the trial, and *K* is the number of data samples. The *RMS* of sEEG or pEEG are denoted as *RMS*_*S*_ or *RMS*_*P*_ and will be used as features in subsequent investigation of the relationship between pain and EEG and in pain prediction.

### Relationship between sEEG and pEEG

To test whether sEEG can serve as a baseline to normalize pEEG for a smaller inter-individual variability, we examined the relationship between *RMS*_*S*_ and *RMS*_*P*_. We assume that an individual's *RMS*_*S*_ and *RMS*_*P*_ (both of which are averaged across all trials at each pain intensity level) are normally distributed, and calculate the mean and standard deviation (SD) of *RMS*_*S*_ and *RMS*_*P*_ across all trials at each pain intensity level. Then, cross-individual correlation between these mean and SD values were estimated.

### Relationship between pain and pEEG

In our experiments, participants were asked to report the level of pain perception with four as the pinprick pain threshold (i.e., NRS > 4 refers to feeling of pinprick pain). Thus, NRS = 4 serves as a threshold to differentiate low-pain and high-pain (i.e., low-pain: NRS ≤ 4, high-pain: NRS > 4). To investigate the relationship between the rating of perceived pain and evoked EEG responses, *RMS*_*P*_, which was averaged across trials with each identical pain level for each individual, was fitted using two models: a global linear model and a two-piecewise linear model (two segments with NRS = 4 as the break point). The global linear model is based on the assumption that magnitude of pEEG is linearly increased with the pain rating, while the piecewise linear model assumes that the relationship between pain rating and pEEG is different for low-pain (NRS ≤ 4) and high-pain (NRS > 4). The performance of the two models are quantified with the mean square error (MSE) and compared across individuals using a paired sample *t*-test.

### Feature normalization based on sEEG

There are different normalization methods available, and here we normalized the magnitude of a pEEG trial as the *z*-score of the population defined by sEEG trials. For each individual, the magnitude of the *i*-th pEEG trial, *RMS*_*P*_(*i*), was normalized by *RMS*_*S*_ of all sEEG trials as

(2) $$\begin{array}{l} nRMS_{P}\left(i\right)=\frac{RMS_{P}\left(i\right)-\mu \left(RMS_{S}\right)}{\sigma \left(RMS_{S}\right)}, \end{array}$$

where *nRMS*_*P*_(*i*) is the normalized magnitude of the *i*-th pEEG trial, μ and σ are respectively the mean and the SD of *RMS*_*S*_ of all trials of this individual.

To examine whether the inter-individual variability of pEEG magnitudes was decreased by the sEEG-based normalization, we performed an ANOVA *F*-test on *RMS*_*P*_ and *nRMS*_*P*_. More precisely, at each pain level, an ANOVA *F*-test was performed on *RMS*_*P*_ or *nRMS*_*P*_ of all trials with this pain level across all individuals to check whether the means of *RMS*_*P*_ or *nRMS*_*P*_ of this group of individuals are the same, and the resultant *F*-statistics denote the inter-individual variability relative to the within-individual variability of the variable under test. Then, we compared the *F*-statistics between *RMS*_*P*_ and *nRMS*_*P*_ at each pain level, and it is expected that *nRMS*_*P*_ has a smaller *F*-statistic than *RMS*_*P*_.

We next investigate whether the proposed sEEG-based normalization method can reduce the cross-individual variability in the relationship between intensity of pain perception and the magnitude of pEEG. Firstly, for each individual, we calculated an optimal threshold of *RMS*_*P*_ or *nRMS*_*P*_ that can best classify (with the highest accuracy) the individual' trials into low-pain (NRS ≤ 4) and high-pain (NRS > 4). The inter-individual variability of the binary classification thresholds of *RMS*_*P*_ and *nRMS*_*P*_ were measured by variance. If the variance of the threshold obtained from *nRMS*_*P*_ is smaller than that from *RMS*_*P*_, the effectiveness of sEEG-based normalization in reducing the individual difference in the binary classification can be validated. A two-sample *F*-test was also conducted to check whether the thresholds of *RMS*_*P*_ and *nRMS*_*P*_ have the same variance. Secondly, we consider the relationship between pain ratings and *RMS*_*P*_ (or *nRMS*_*P*_) of high-pain (NRS > 4) trials to be a linear model specific to each individual, then the inter-individual variability in this relationship is indicated by the cross-individual variance of slope and intercept of the linear model. So, slopes and intercepts of all individuals were calculated using two sets of features (*RMS*_*P*_ and *nRMS*_*P*_), and their inter-individual variability were compared. If the cross-individual variance of slopes or intercepts obtained using *nRMS*_*P*_ is smaller than that obtained using *RMS*_*P*_, the effectiveness of sEEG-based normalization in reducing the individual variability in the continuous prediction model can be validated. Similarly, a two-sample *F*-test was also conducted to check whether the slopes or intercepts of *RMS*_*P*_ and *nRMS*_*P*_ have the same variance.

### Binary classification (low-pain vs. high-pain)

A linear discriminant analysis (LDA) classifier was adopted to classify low-pain and high-pain trials using leave-one-individual-out cross validation. The classifier was first trained with *RMS*_*P*_ or *nRMS*_*P*_ of training trials, which divided into two categories (low-pain: NRS ≤ 4, and high-pain: NRS > 4), and then applied to the test trials to predict labels (low-pain vs. high-pain) from the corresponding *RMS*_*P*_ or *nRMS*_*P*_. The classification performance was evaluated by accuracy, and the accuracies obtained from classification using *RMS*_*P*_ and *nRMS*_*P*_ were compared using paired sample *t*-test.

### Continuous prediction of pain levels for high-pain trials

After binary classification, only high-pain trials are involved in continuous pain prediction, because there is no significant correlation between pain ratings and pEEG of low-pain trials. To prove that the sEEG-based normalization is effective for continuous pain prediction regardless of the results of the preceding binary classification, we performed continuous pain prediction for trials predicted as high-pain from binary classification as well as for real high-pain trials (NRS > 4).

Relationship between single-trial *RMS*_*P*_ (or *nRMS*_*P*_) and the corresponding intensity of pain perception was modeled by linear regression. For the *i*-th pEEG trial, the pain rating *R*_*i*_ can be estimated as

(3) $$\begin{array}{r} R_{i}=\alpha \cdot RMS_{P}\left(i\right)+c, \end{array}$$

(4) $$\begin{array}{r} R_{i}=\alpha \cdot nRMS_{P}\left(i\right)+c, \end{array}$$

where α and *c* are slope and intercept of the linear regression model. The model of (Equations 3 and 4) was trained and tested using leave-one-individual-out cross validation.

The prediction performance of the linear regression model was evaluated by Mean Absolute Error (MAE),

(5) $$\begin{array}{l} MAE=\frac{1}{N}\overset{N}{\underset{n=1}{\sum }}|R_{i}-{\hat{R}}_{i}|, \end{array}$$

where *N* is the total number of test trials, *R*_*i*_ and ${\hat{R}}_{i}$ are respectively the real and predicted rating value for the *i*-th trial. MAE provides a straightforward measure on how precisely the generated linear regression model can represent the relationship between pain ratings and pEEG magnitudes. The MAE values obtained from prediction using *RMS*_*P*_ and *nRMS*_*P*_ were compared using the paired sample *t*-test.

## Results

### Relationship between sEEG and pEEG

**Figure 1:**
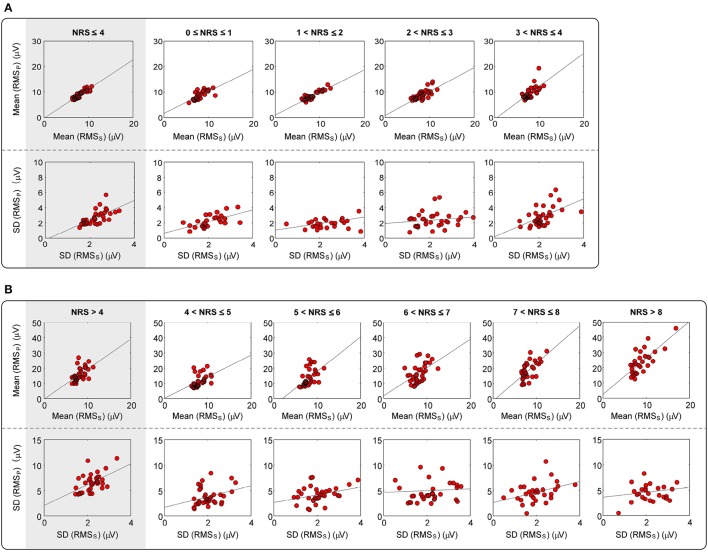
**Correlation between the mean and SD of ***RMS***_***S***_ and ***RMS***_***P***_ at levels of (A) NRS ≤ 4, and (B) NRS > 4**. Red dots represent the mean or SD of *RMS*_*S*_ and *RMS*_*P*_, which are averaged across all trials at each pain intensity level for each participant. Gray lines represent the best linear fit.

For each participant, the mean and SD of *RMS*_*S*_ and *RMS*_*P*_ at each pain intensity level were calculated. Since NRS > 8 was not available for some participants, we use a combined level of “NRS > 8” to denote all trials with an NRS > 8. It can be clearly seen from Figure 1 and Table 1 that, a significant correlation (*p* ≤ 0.007) between the mean values of *RMS*_*S*_ and *RMS*_*P*_ was obtained at each intensity level of pain perception. In addition, a significant correlation between the SD values of *RMS*_*S*_ and *RMS*_*P*_ was also obtained (*p* ≤ 0.02) for overall intensity level of low-pain (NRS ≤ 4) and high-pain (NRS > 4), though some individual intensity level is not significant (such as intensity level at 2–3, 4–5, 6–7, and 8–10). To conclude, the distributions of *RMS*_*S*_ and *RMS*_*P*_ are highly correlated, which verifies our hypothesis that magnitudes of pEEG are highly correlated with the magnitude of sEEG. This observation supports the idea that *RMS*_*S*_ could serve as an individual scale to normalize his/her *RMS*_*P*_ to reduce inter-individual variability of pain-related features in pain classification and prediction models.

**Table 1:** **Correlation between the mean and SD of ***RMS***_***S***_ and ***RMS***_***P***_ at each pain level**.

**NRS**		**(0, 4]**	**(0, 1]**	**(1, 2]**	**(2, 3]**	**(3, 4]**	
Mean	*R*	0.891	0.721	0.930	0.725	0.666	
	*P*-value	< 0.001	< 0.001	< 0.001	< 0.001	< 0.001	
SD	*R*	0.659	0.614	0.466	0.171	0.537	
	*P*-value	< 0.001	< 0.001	0.013	0.335	0.001	
**NRS**		**(4**, **10]**	**(4**, **5]**	**(5**, **6]**	**(6**, **7]**	**(7**, **8]**	**(8**, **10]**
Mean	*R*	0.550	0.454	0.525	0.570	0.633	0.555
	*P*-value	< 0.001	0.007	0.001	< 0.001	< 0.001	0.003
SD	*R*	0.519	0.303	0.340	0.065	0.402	0.193
	*P*-value	0.002	0.082	0.049	0.718	0.025	0.346

### Relationship between pain and pEEG

**Figure 2:**
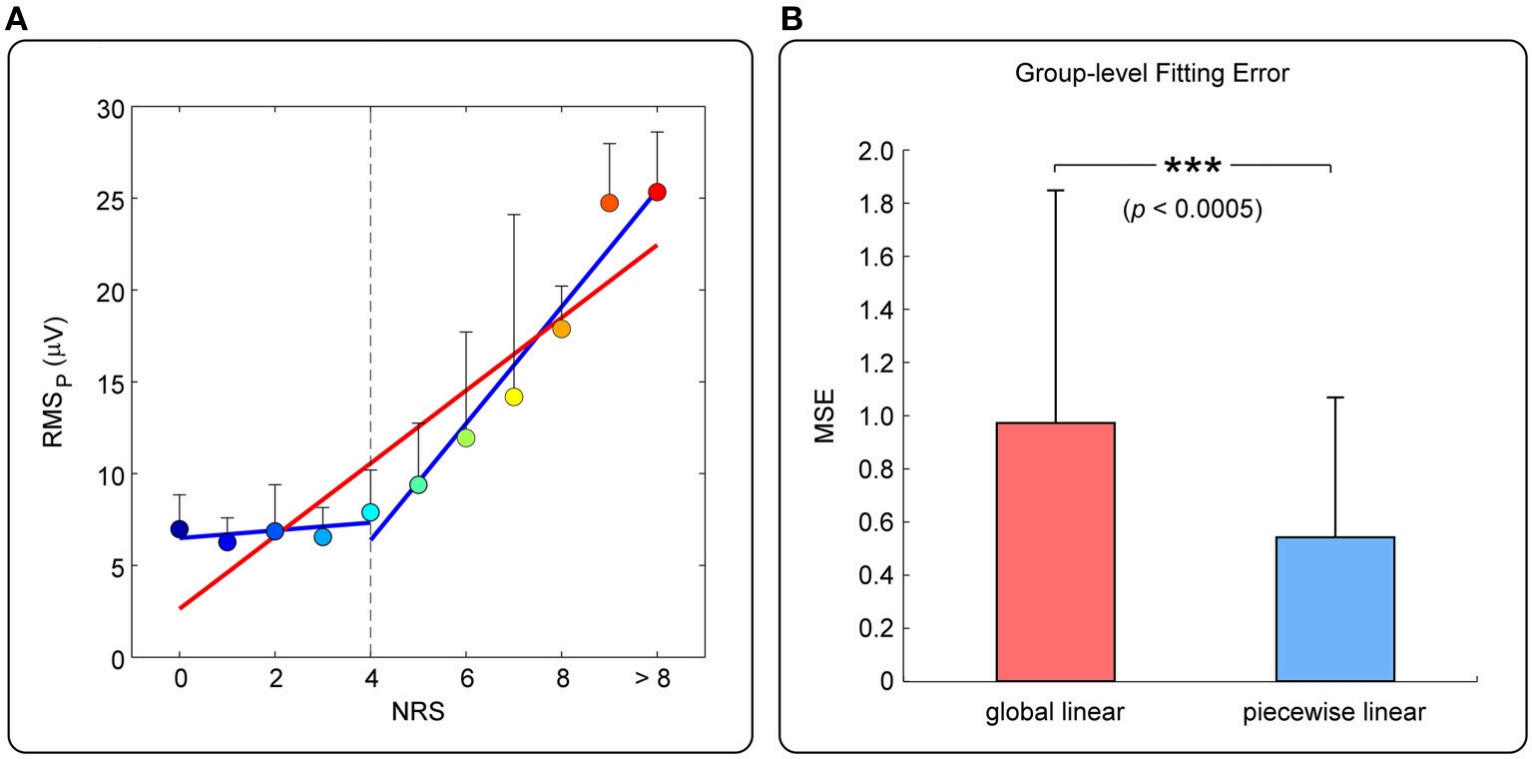
**(A)** Relationship between pain ratings and *RMS*_*P*_ (from one participant). Colored dots represent mean ± SD of *RMS*_*P*_ averaged across trials at different level of pain perception. The red line represents the fitted global linear model, while the blue lines represent the fitted two-piecewise linear model. **(B)** Comparison of MSE (mean ± SD) of all participants between two fitting models.

Figure 2A shows the relationship between pain rating and the magnitude of pEEG (mean ± SD) of one participant. Overall, pain rating and *RMS*_*P*_ are positively related, but *RMS*_*P*_ does not increase significantly when the subjective pain ratings is ≤ 4 (referred to as “low-pain”); when the subjective pain rating is >4 (referred to as “high-pain”), *RMS*_*P*_ is linearly increased with pain ratings. MSE of the global linear model (red line) or the two-piecewise linear model (blue line) was adopted to measure the accuracy of fitting, as shown in Figure 2B. It can be seen from the group-level results in Figure 2B that, the fitting error of the piecewise linear model is significantly smaller than that of the global linear model. Therefore, the piecewise linear model can better describe the relationship between pain perception and *RMS*_*P*_. The nonlinear relationship motivates us to develop the two-stage pain prediction (i.e., to classify low- and high-pain first, then to predict the pain rating for high-pain trials only).

### Feature normalization based on sEEG

We first confirmed that the magnitudes of sEEG trials and pEEG trials of each individual approximately follow a normal distribution (*p* < 0.0001 for all individuals, one-sample Kolmogorov–Smirnov test). Therefore, an individual's sEEG trials could form a distribution for normalizing pEEG trials into *z*-scores. Table 2 shows that, at each pain level, the *F*-statistic obtained from the ANOVA *F*-test on *nRMS*_*P*_ is lower than that obtained from *RMS*_*P*_, which proves that the sEEG-based normalization method can effectively reduce the inter-individual variability of pEEG trials.

**Table 2:** **Comparison of *F*-statistics obtained from the ANOVA *F*-test on *RMS*_*P*_ and *nRMS*_*P*_**.

**NRS**	**(0, 1]**	**(1, 2]**	**(2, 3]**	**(3, 4]**	**(4, 5]**	**(5, 6]**	**(6, 7]**	**(7, 8]**	**(8, 10]**
*RMS_*P*_*	5.279	6.476	5.802	10.553	17.022	13.501	17.278	10.420	18.718
*nRMS_*P*_*	1.182	1.475	1.582	4.795	11.624	11.332	13.792	8.172	9.310

**Figure 3:**
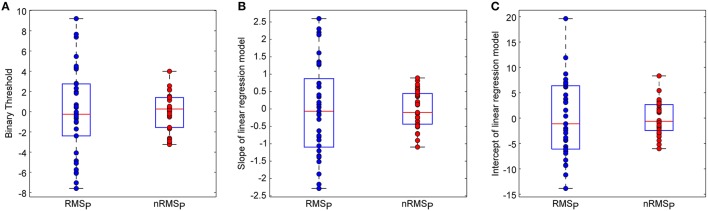
**Effects of sEEG normalization on inter-individual variability of (A) binary classification thresholds, (B) slopes of linear regression models for high-pain trials, (C) intercepts of linear regression models for high-pain trials**. Noted that mean values were removed from these parameters for illustration. The box plots show the minimu, lower quartile, median, upper quartile, and maximum values of one group of variables.

Figure 3 shows the optimal binary classification thresholds and slopes/intercepts of linear regression models for high-pain trials, which were obtained from available trials and ratings of all individuals. We can clearly see that, after sEEG-based normalization, the variance of all above three parameters were remarkably decreased, which illustrates that the proposed sEEG-based normalization method can effectively reduce the inter-individual variability in classification and prediction models.

Table 3 further shows that, cross-individual variances of all three classifier/model parameters were decreased after sEEG-based normalization. The two-sample *F*-test also demonstrates that the variances of all three classifier/model parameters are significantly different between using *RMS*_*P*_ and using *nRMS*_*P*_ as features (*p* < 0.0001 for all).

**Table 3:** **Comparison of binary classifier thresholds and model parameters between using ***RMS***_***P***_ and using ***nRMS***_***P***_**.

		***RMS_*P*_***	***nRMS_*P*_***
Binary classfication theresholds	Cross-individual variance	4.284	1.797
	*P*-value (*F*-test for equal variance)	< 0.001	
Slopes of linear models	Cross-individual variance	1.340	0.522
	*P*-value (*F*-test for equal variance)	< 0.001	
Intercepts of linear models	Cross-individual variance	7.525	3.254
	*P*-value (*F*-test for equal variance)	< 0.001	

### Pain prediction

The mean and SD of accuracy for binary pain classification (low-pain vs. high-pain) using *RMS*_*P*_ (i.e., pEEG features) and using *nRMS*_*P*_ (i.e., sEEG-normalized pEEG features) are summarized in Table 4. Results show that *nRMS*_*P*_ can improve the classification accuracy, though the performance improvement is only close to significant (*p* = 0.092).

**Table 4:** **Accuracy of binary classification and prediction error (MAE) of continuous prediction**.

	***RMS_*P*_***	***nRMS_*P*_***	***P*-value (paired *t*-test)**
Accuracy of binary classification (%)	68.95 ± 12.91	70.36 ± 14.18	0.092
MAE of continuous prediction (on predicted high-pain trials)	1.838 ± 0.602	1.625 ± 0.446	0.002
MAE of continuous prediction (on real high-pain trials)	1.235 ± 0.278	1.173 ± 0.278	0.003

The mean and SD of MAE for continuous pain prediction using *RMS*_*P*_ (i.e., pEEG responses) and using *nRMS*_*P*_ (i.e., sEEG-normalized pEEG responses) for trials predicted as high-pain from binary classification and for real high-pain trials (NRS > 4) are summarized in Table 4. Results indicate that the proposed sEEG-based normalization method can significantly improve the prediction accuracy for both predicted high-pain trials (*p* = 0.002) and real high-pain trials (*p* = 0.003) in continuous pain prediction.

## Discussion

In this study, we proposed to normalize pain-evoked EEG responses using spontaneous EEG to improve the performance of EEG-based pain prediction. Pain-related EEG responses have been used to predict the level of subjective pain, but the large inter-individual variability seriously degrades the performance of cross-individual pain prediction. In this work, we began by performing a comprehensive and detailed investigation of the relationship between pEEG responses and sEEG activities as well as the relationship between subjective pain ratings and pEEG responses. Our results revealed a strong inter-individual correlation between the magnitude of pEEG and sEEG. Besides, our results also confirmed a nonlinear relationship between pEEG and subjective pain ratings. Based on above observations, we proposed a new two-stage approach for pain prediction: (1) a binary classification to differentiate low-pain and high-pain trials; (2) a continuous regression to predict pain ratings of high-pain trials. In both steps, the normalization strategy based on sEEG was used to reduce the inter-individual variability in the magnitude of pEEG, so that a higher classification accuracy and a lower prediction error were achieved. The new sEEG-based normalization strategy has the potential to contribute to an applicable and reliable tool for pain assessment.

### Relationship between sEEG and pEEG

An individual's spontaneous EEG has been shown to be related to his/her genetic code, implying its uniqueness (Tran et al., 2001; Doležal et al., 2005; Anokhin et al., 2006; Marcel and Del Millan, 2007; Näpflin et al., 2007; Zietsch et al., 2007). A strong inter-individual correlation between the magnitude of sEEG and pEEG was also obtained from our database. A potential interpretation for this phenomenon may be due to the skull thickness, the orientation of the gray matter and so forth. These anatomical factors are specific to each person and will remain relatively stable for adults. Experimental conditions, such as electrode position and scale-electrode impedances, could also contribute to the phenomenon, because they may influence the magnitudes of both pEEG and sEEG.

### Relationship between pain rating and pain-evoked EEG responses

Numerous previous studies have shown that the perceived pain intensity is strongly correlated with the amplitude of a number of evoked EEG responses (Iannetti et al., 2005; Huang et al., 2013b). In most of these works, the level of pain perception was assumed or found to be linearly correlated with the evoked EEG responses, but such a linear relationship has been challenged by growing evidence showing the nonlinearity between pain level and the neural responses. The assumption and observation of the linear relationship may due to the limited range of painful stimulus intensities used in most of pain experiments, which further limited the range of perceived pain intensity. To solve this problem, our experiment was designed to deliver a large number of laser pulses (>100) with a wide energy range (from 1 to 3.75–4.5 J; 12−15 levels) to each participant. The result that the fitting error of a two-piecewise linear model (with a break point of NRS = 4) was significantly smaller than that of a global linear model indicated a nonlinear relationship between pain level and the evoked EEG responses.

### Feature normalization based on sEEG

To normalize the magnitudes of pEEG trials of one individual, we proposed to estimate their *z*-scores in the population defined by sEEG trials of this individual. Although other normalization methods exist, the proposed *z*-scores can achieve better prediction results than other normalization methods, such as dividing *RMS*_*P*_ with the mean of sEEG or subtracting the mean of sEEG from *RMS*_*P*_ (results are not shown here). Besides its good performance, the proposed *z*-score normalization also reflects certain physiological meanings. It has been revealed that the variability of spontaneous neural activity can reflect the “dynamic range” of possible neural responses to incoming stimuli and can provide a powerful and accessible measure for understanding various individual difference variables (Barlow, 1960; Rodin et al., 1965; Rogers, 1980; Polich, 1997; Ramos-Loyo et al., 2004; Lee et al., 2011; Nash et al., 2012; Garrett et al., 2013; Schiller et al., 2014). Therefore, the distribution defined by the magnitudes of sEEG is indicative of the possible range of magnitudes of evoked pEEG and it can be used as a baseline distribution to normalize pEEG magnitudes to *z*-scores.

### Applicability of the sEEG-normalization based pain prediction

Predictive power of pEEG responses for decoding the intensity of subjective pain perception has been well documented in previous studies (Kakigi et al., 1989; Bromm and Treede, 1990; Garcí-Larrea et al., 1997; Iannetti et al., 2005; Huang et al., 2013b), which further led to several cross-individual pain prediction methods, which do not need any subjective pain rating for new individuals and thus more promising for clinical uses. However, the accuracy of cross-individual pain prediction is still not satisfactory because of the inherent inter-individual variability in either pain evoked responses or pain ratings. A practical solution to this problem is to incorporate individual traits that are related to inter-individual variability into the pain prediction model (Davis, 2011). In our previous study (Huang et al., 2013b), single-trial evoked EEG features were normalized by subtracting the mean and dividing by the SD of the individual's evoked EEG features, and single-trial ratings of pain perception were rescaled within the range from 0 to 10 (defining 0 as the lowest pain rating and 10 as the highest pain rating for each participant). Although above normalization on both evoked EEG features and pain ratings can significantly increase the prediction accuracy, its drawback was obvious. First, the normalization was based on the distribution of evoked EEG features, which can only be obtained from a large number of painful stimuli and may not be accepted by participants. Second, it still needs subjective pain rating from a new individual, which is not suitable for participants with communication impairments. As compared with the conventional normalization strategy (Huang et al., 2013b), the proposed method has two main advantages: first, it will not introduce any pain experience to a new participant because the normalization is based on spontaneous EEG; second, it can well deal with the difficult situation that no reliable pain rating is available because no subjective rating is needed. Therefore, the proposed sEEG-based normalization method is more practical and feasible for clinical research and applications.

### Limitation and future work

The proposed normalization strategy focused solely on features of pain-evoked EEG responses, simply because real values of pain perception are generally considered to be unknown in clinical scenarios. However, not only EEG responses but also the pain ratings are characterized by tremendous inter-individual variability. Different individuals perceive different pain perception in response to the same painful stimulus. For example, we have found pronounced sex-dependent difference in pain perception as well as in pain-evoked EEG responses (see Supplementary Materials). Taking into account gender difference (such as using sex as a predictor) may lead to a more accurate pain prediction. Mechanisms contributing to inter-individual differences in pain sensitivity include genetic, environmental, psychological, and cognitive factors (Nielsen et al., 2009; Coghill, 2010; Schulz et al., 2012), and it may be caused at any stage in pain processing from the skin to the brain. Highly sensitive individuals may activate stronger neural responses and/or pain experience than insensitive individuals (Coghill et al., 2003; Coghill, 2010). Variations in pain sensitivity is an important issue worthy of further investigation, because understanding the contributing factors of pain sensitivity will help greatly in developing a more accurate and practical method for diagnosis of pain (Edwards, 2005; Nielsen et al., 2009). Our future study is aimed to address above difficult problems, such as how to normalize pain ratings and pain sensitivity and how to incorporate personal traits and environmental factors in the prediction model, to develop a more accurate and practical EEG-based prediction assessment method.

## Author contributions

YB and ZZ designed the study. YB, GH, and AT collected the data. YB, YT, and GH analyzed the data. YB, GH, YT, AT, YH, and ZZ discussed the results and wrote the paper.

### Conflict of interest statement

The authors declare that the research was conducted in the absence of any commercial or financial relationships that could be construed as a potential conflict of interest.
