# Supplementary material for: Normalization of Pain-Evoked Neural Responses Using Spontaneous EEG Improves the Performance of EEG-Based Cross-Individual Pain Prediction
Source: Front Comput Neurosci. 2016 Apr 13;10:31. doi: 10.3389/fncom.2016.00031 (PMC4829613; doi:10.3389/fncom.2016.00031)
Supplement: Supplementary file 1 [file Presentation1.PDF]

Supplementary Materials

Firstly, we examined the difference of pain ratings with same stimulus energy (14 levels from 1 – 4.25 J, in step of 0.25 J) between males and females, as shown in Fig. S1 (A). It can be seen from Table S1 that significant sex-dependent difference was observed at most of the stimulus levels (except levels 3, 12, and13). Next, a similar analysis was conducted to compare RMS_P values with same rating values (NRS from 0 – 8) between males and females, as shown in Fig. S1 (B). Statistical analysis in Table S2 illustrated significant sex-dependent difference at every rating value. Please note that all above differences were obtained using two sample t-test.

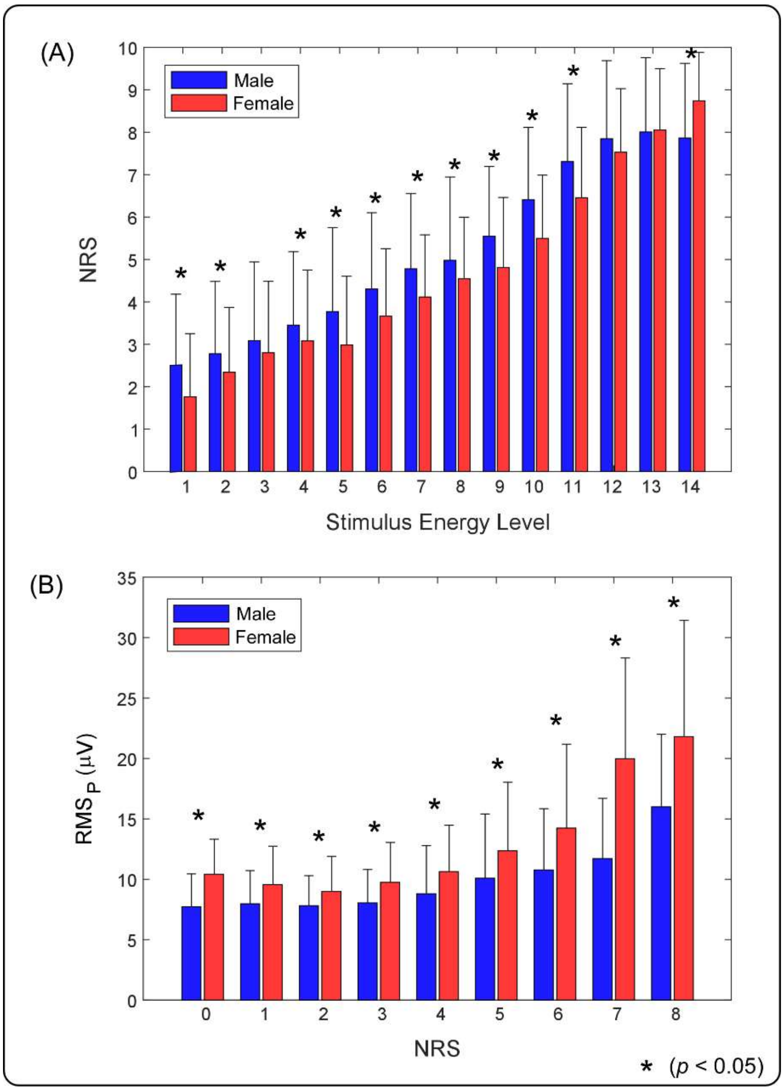

Figure S1. (A) Difference in pain ratings with same stimulus energy between males and females. (B) Difference in RMS_P with same rating value between males and females.

Table S1. P-values of comparisons of pain ratings with same stimulus energy between males and females

**Level of Stimulus Energy**	1	2	3	4	5	6	7
**P-value**	2.3e-5	0.014	0.15	0.046	1.2e-4	6.4e-4	2.7e-4
**Level of Stimulus Energy**	8	9	10	11	12	13	14
**P-value**	0.026	8.2e-5	6.0e-7	2.9e-5	0.11	0.84	0.038

Table S2. P-values of comparisons of RMS_P with same rating value between males and females

**NRS**	0	1	2	3	4	5	6	7	8
**P-value**	3.0e-6	5.7e-6	5.7e-5	6.8e-8	1.4e-9	2.5e-7	6.9e-8	<1e-9	<1e-9

The results showed significant sex-dependent difference in pain perception as well as in pain-evoked EEG responses, and the findings are consistent with the literatures (Eli et al., 2000; Fillingim, 2000; Riley et al., 1998; Wiesenfeld-Hallin, 2005).

Reference

Eli, I., Baht, R., Kozlovsky, A., and Simon, H. (2000). Effect of gender on acute pain prediction and memory in periodontal surgery. *European Journal of Oral Sciences* 108**,** 99-103.

Fillingim, R.B. (2000). Sex, gender, and pain: Women and men really are different. *Current Review of Pain* 4**,** 24-30.

Riley, J.L., 3rd, Robinson, M.E., Wise, E.A., Myers, C.D., and Fillingim, R.B. (1998). Sex differences in the perception of noxious experimental stimuli: a meta-analysis. *Pain* 74**,** 181-187.

Wiesenfeld-Hallin, Z. (2005). Sex differences in pain perception. *Gender Medicine* 2**,** 137-145.
